# Heat stress induces intestinal injury through lysosome- and mitochondria-dependent pathway *in vivo* and *in vitro*

**DOI:** 10.18632/oncotarget.16580

**Published:** 2017-03-28

**Authors:** Gao Yi, Li Li, Meijuan Luo, Xuan He, Zhimin Zou, Zhengtao Gu, Lei Su

**Affiliations:** ^1^ Southern Medical University, Guangzhou, 510515, P.R. China; ^2^ The Fifth Affiliated Hospital of Guangzhou Medical University, Guangzhou, 510700, P.R. China; ^3^ Department of Intensive Care Unit, The Third Affiliated Hospital of Southern Medical University, Guangzhou 510630, P.R. China; ^4^ Department of Pathophysiology, Southern Medical University, Guangdong Provincial Key Laboratory of Shock and Microcirculation Research, Guangzhou 510515, P.R. China; ^5^ Department of Pediatrics, Guangzhou First People's Hospital, Guangzhou Medical University, Guangzhou, 510180, P.R. China; ^6^ Department of Intensive Care Unit, Guangzhou General Hospital of Guangzhou Military Command, Guangzhou, 510010, P.R. China

**Keywords:** heat stress, cathepsin B, lysosomal membrane permeabilization, mitochondrial, apoptosis

## Abstract

Damage to the small intestine secondary to heat stroke is a major factor in heat stroke-related morbidity and mortality. However, the underlying mechanisms by which heat stroke causes small intestinal lesions and dysfunction remain unclear. To explore the pathogenesis of small intestinal tissue and epithelial cell injury, the SW480 cell heat stress model and the mice heat stroke model were established to mimic heat stroke. Morphologic changes in intestinal tissue and increased TUNEL-positive index were induced by heat stress *in vivo*. Heat stress activated the lysosomal-mitochondrial apoptotic pathway in SW480 cells, increasing intracellular reactive oxygen species and causing lysosomal membrane permeabilization with subsequent release of cathepsin B to the cytosol, mitochondrial depolarization, and cytochrome C release to cytosol. An increase in the Bax/Bcl2 ratio, caspase-9 and caspase-3 were observed. N-Acetyl-L-Cysteine was shown to inhibit ROS generation, suppress permeabilization of lysosomal membranes, decrease levels of cathepsin B and cytochrome C in the cytosol, and inhibit Bax/Bcl2 ratio, caspase-9 and caspase-3 activity both *in vitro* and *in vivo*. Mitochondrial damage was alleviated when the models were pre-treated with CA-074 Me both *in vitro* and *in vivo*, decreasing cathepsin B and cytochrome C levels in the cytosol, Bax/Bcl2 ratio, caspase-9 and caspase-3 activity. In our models, heat stress-induced apoptosis of small intestinal tissue and epithelial cells through accumulation of ROS and activation of the lysosomal–mitochondrial apoptotic pathway involved the release of cathepsin B. These findings may offer potentially pharmaceutical targets and strategies to repair intestinal injury caused by heat stroke.

## INTRODUCTION

Heat stress is a common stressor that affects many biological systems [1, 2]. Heat stroke can be fatal if not urgently and appropriately managed [3]. When an individual is exposed to high ambient temperatures, heat stroke can affect vital organs; one of the primary organs affected by heatstroke is the gastrointestinal tract [4]. Multiple studies have pathologically demonstrated damage to the small intestine following heat stress [3, 5, 6]. Previously, we reported on heat stroke causing injury to the small intestine and impairing the integrity of the intestinal barrier and intestinal permeability [3, 7].

Apoptosis is the physiological mechanism by which cells die under strict control [5, 6]. Heat stress may serve as an inducer of cellular apoptosis [7–9]. When an individual is exposed to high temperature, the body restricts blood flow to the small intestine to preserve blood flow to essential organs, such as the brain and the heart. The reduction in gastrointestinal blood flow greatly impairs the epithelial cells of the small intestinal villi and induces excessive apoptosis [5, 10, 11]. Recently, studies in multiple animal models have detected heat stress-induced apoptosis in small intestinal tissue and intestinal epithelial cells [5, 12–14]. However, little is known about the biological mechanisms involved in heat stress-induced apoptosis in the small intestine.

Lysosomes are membrane-bound organelles that contain an arsenal of different hydrolases, enabling them to act as the terminal degradative compartment of the endocytotic, phagocytic and autophagic pathways [15, 16]. Several *in vivo* and *in vitro* modelshave elucidated the role of lysosomes and lysosomalenzymes in initiating and executing the apoptotic program, and the existence of ‘lysosomal pathway apoptosis’ is generally accepted [17–19]. About a decade ago, the “lysosomal–mitochondrial axis” apoptosis signal pathway was proposed, suggesting an interplay between the lysosomal apparatus and mitochondria in apoptosis [15, 20]. The lysosomal-mitochondrial apoptotic pathway is initiated by a critical step when the lysosomal membrane becomes permeable and releases lysosomal contents into the cell cytosol. Cathepsins, especially cathepsin B, are proteases released from the lysosome into the cytosol during this process and have been found to participate in apoptosis. Cathepsin B cleaves Bid and degrades anti-apoptotic Bcl-2 proteins in the cytosol, leading to an activation of caspases and subsequent mitochondrial depolarization. This process triggers the mitochondrial pathway of apoptosis [15, 20].

A survey of the available literature reveals that numerous agents and molecules of endogenous or synthetic origin can induce lysosomal membrane permeabilization (LMP) [15, 21]. Among these agents, the reactive oxygen species (ROS) are arguably one of the most important endogenous LMP inducers [15]. Small bowel ischemia has been found to promote ROS formation. The increase in free radicals from ischemia can lead to ROS-related damage of the intestinal mucosa. Heat stress-related oxidative stress has been shown to cause apoptosis in the small intestines of both rats and pigs [5, 13, 14]. Therefore, oxidative stress may be the intermediate through which heat stress induces intestinal damage and may act upstream of apoptosis. However, the precise molecular mechanism by which heat stress induces ROS and subsequent apoptosis in small intestinal tissue and small intestinal epithelial cells is still poorly understood.

Due to the phenomenon of global warming, ambient temperatures and morbidity from heat stroke are predicted to increase over the next decades. Consequently, better understanding of the molecular mechanisms underlying cellular changes from heat stress is needed. And recently, there are many studies have determined that ROS acts as an up-streams in lysosomal injury and mitochondrial apoptotic pathway in SW480 cells by various different stimulating factors [22–24]. This study sought to identify factors involved in the pathophysiology of heat stress-induced injury in the small intestine *in vitro* and *in vivo*. A heat stress model using SW480 cells and a heat stroke mouse model were established. We hypothesized that ROS act as an upstream regulator of cathepsin B in the lysosomal-mitochondrial apoptotic pathway induced by heat stress both *in vitro* and *in vivo*.

## RESULTS

### Effects of heat stress on cell viability and cytotoxicity in SW480 cells

**Figure 1:**
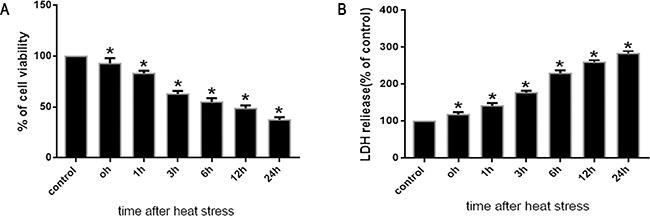
Effects of heat stress on cell viabilityand cytotoxicity in SW480 cells SW480 cells underwent intense heat stress (43°C) for 2h, and were further incubated at 37°C for different times as indicated (0h, 1h, 3h, 6h, 12h or 24h). The percentages of viable cells and dead cellswere assessed by WST-1 **A**. and LDH release **B**. assays. Percent viability is expressed relative to control cells cultured at 37°C. The data shown represent the mean ±SD of at least three independent experiments, performed in duplicate. *P < 0.05, statistically significant relative to control (37°C).

To investigate changes in cell viability and cytotoxicity following heat stress in SW480 cells exposed to heat stress (43°C), both the WST-1 and LDH assays were used. SW480 cells were maintained in standard culture media for 48h at 37°C prior to a temperature shift to intense heat stress (43°C) for 2h, and were further incubated at 37°C for different times as indicated (0h, 1h, 3h, 6h, 12h and 24h). After cells were heat stressed and cultured, cell viability was shown to decline drastically concomitant with a significant increase in cytotoxicity, as indicated by the time-dependent reduction in formazan formation and increase in LDH activity, respectively (Figure 1A, 1B).

### Heat stress-induced increasing of ROS in SW480 cells

**Figure 2:**
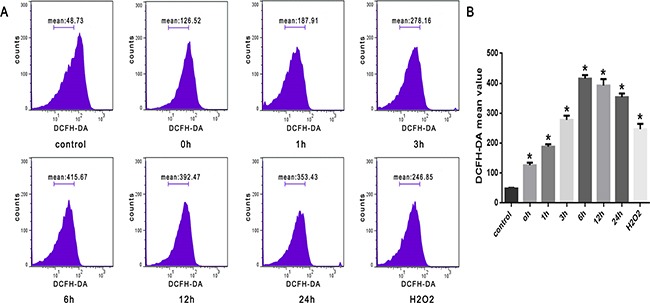
ROS increased after heat stress in SW480 cells SW480 cells underwent intense heat stress (43°C) for 2h, and were further incubated at 37°C for different times as indicated (0h, 1h, 3h, 6h, 12h or 24h). Cells were labeled with 10 μM DCFH-DA for detection of ROS. H_2_O_2_ was used as a positive control. **A**. Flow cytometry analysis of heat stress-induced ROS. **B**. The histogram represents quantified ROS generation after heat stress analyzed by flow cytometry. The data shown represent the mean ±SD of at least three independent experiments, performed in duplicate. *P < 0.05, statistically significant relative to control (37°C).

As shown in Figure 2, we observed a time-dependent increase in intracellular ROS levels in SW480 cells that were treated with heat stress (43°C). There was a noticeable increase immediately at 0h that peaked at 6h after heat stress.

### Lysosomal membrane permeabilization induced by heat stress in SW480 cells

**Figure 3:**
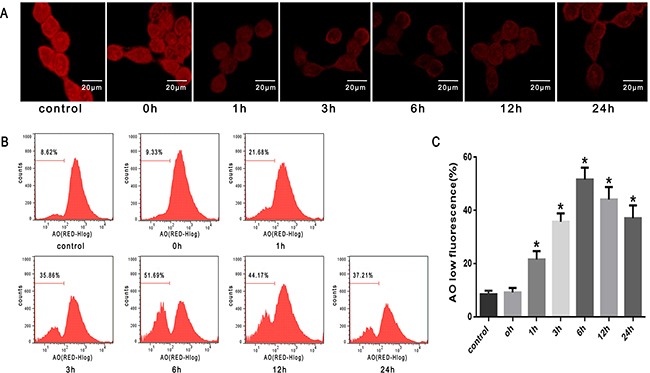
Lysosomal membrane permeabilization induced by heat stress in SW480 cells SW480 cells underwent intense heat stress (43°C) for 2h and were further incubated at 37°C for different times as indicated (0h, 1h, 3h, 6h, 12h or 24h). Cells were labeled with5 μM acridine orange (AO). **A**. Confocal laser scanning microscopy images of fluorescently labeled cells (×400). **B**. Flowcytometry analysis to count cells with a reduced number of intact lysosomes (‘pale cells’). **C**. The histogram represents the quantification of ‘pale cells’ analyzed by flow cytometry after heat stress. The data shown represent the mean ±SD of at least three independent experiments, performed in duplicate. *P < 0.05, statistically significant relative to control (37°C).

Fluorescent probes-AO are metachromatic fluorophores that yield red fluorescence when they accumulate within the lysosomes. Exposure of SW480 cells to heat stress (43°C) led to a time-dependent decrease in red fluorescence detected by confocal laser scanning microscopy, which sharply decreased at 1h after heat stress (Figure 3A). Furthermore, we used flow cytometry analysis to count cells with a reduced number of intact lysosomes (‘pale cells’); the increase in the percentage of ‘pale cells’ was also time-dependent and increased drastically at 1h after heat stress (Figure 3B, 3C).

### Heat stress activated cathepsin B release to cytosol in SW480 Cells

**Figure 4:**
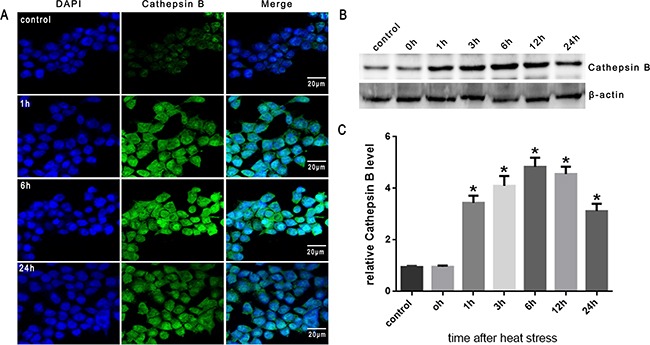
Heat stress activates cathepsin B release to cytosol in SW480 Cells SW480 cells underwent intense heat stress (43°C) for 2h and were further incubated at 37°C for different times as indicated (0h, 1h, 3h, 6h, 12h or 24h). **A**. Localization of cathepsin Bwas visualized by immunofluorescence using a confocal microscope (×200). **B**. Expression of cathepsin B was determined by Western blot. β-actin was run as an internal control. **C**. Quantification of Western blots for cathepsin B after heat stress. Graphs represent mean±SD of at least three independent experiments. *P < 0.05, compared with control group (37°C).

It has been demonstrated that lysosomal destabi-lization is followed by the release of lysosomal cathepsin proteases into the cytosol; cathepsin B, a cysteine protease, has been indicated as a necessary mediatorinthe activation of downstream events leading to cell death [15, 17]. As shown in Figure 4A, cathepsin B fluorescence was punctate and mainly perinuclear in untreated cells. However, following heat stress (43°C) for 2h, cathepsin B became progressively diffuse as it was released from lysosomes into the cytosol when incubated at 37°C for different times as indicated (1h, 6h and 24h). Western blot analysis also indicated that heat stress caused cathepsin B release into the cytosol at 1h, peaked at 6h, and continued up to 24h after heat stress (Figure 4B, 4C).

### Heat stress activates apoptosis of SW480 cells through the mitochondrial pathway

**Figure 5:**
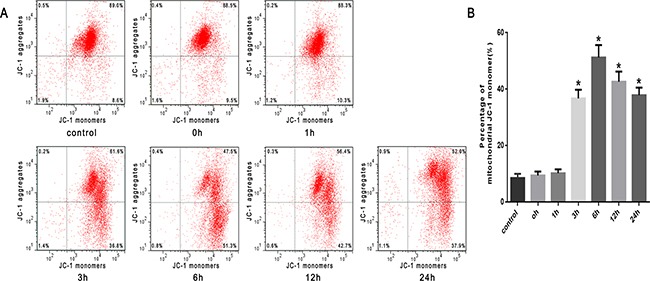
Heat stress induces mitochondrial depolarization (low *ΔΨ*m) in SW480 Cells SW480 cells underwent intense heat stress (43°C) for 2h and were further incubated at 37°C for different times as indicated (0h, 1h, 3h, 6h, 12h or 24h). Cells were labeled with5 μmol/L JC-1. **A**. Flow cytometry analysis of heat stress-induced mitochondrial depolarization (low *ΔΨ*m). **B**. The histogram represents the quantification of the percentage of low *ΔΨ*m analyzed by flow cytometry after heat stress. The data shown represent the mean ±SD of at least three independent experiments, performed in triplicate. *P < 0.05, statistically significant relative to control (37°C).

To investigate mitochondrial function, mitochondrial depolarization (low *ΔΨ*m) was assayed and expressed as a change in JC-1 fluorescence from red to green. We found that the proportion of SW480 cells with low *ΔΨ*m increased from 8.4% in untreated cells to 36.8% in cells heat stressed for 3h, and further increased to 51.3% in cells heat stressed for 6h. A subsequent decrease in *ΔΨ*m to 42.7% and 37.9% was seen at 12h and 24h after heat stress, respectively (Figure 5A, 5B).

**Figure 6:**
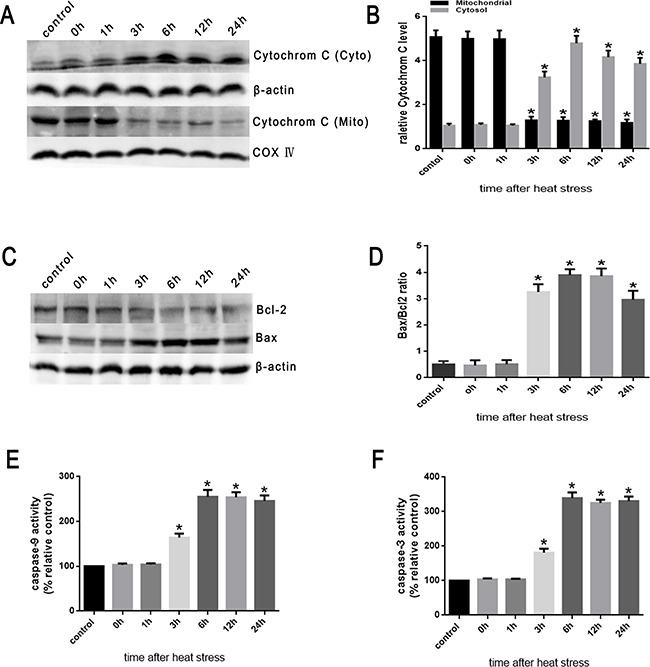
Heat stress induces the activation of mitochondrion-associated pro-apoptotic proteins in SW480 Cells SW480 cells underwent intense heat stress (43°C) for 2h and were further incubated at 37°C for different times as indicated (0h, 1h, 3h, 6h, 12h or 24h). **A**. Intracellular location of cytochrome C was determined by Western blot. β-actin was run as an internal control. COX IV was used as a mitochondrial loading control. **B**. Quantification of Western blots for cytochrome C after heat stress. **C**. Bcl-2 and Bax were determined by Western blots. β-actin was run as an internal control. **D**. Quantification of Western blots for Bax/Bcl-2 ratio after heat stress. **E**. Enzymatic activity of caspase-9 was measured in cell lysates using a fluorogenic substrate, Ac-LEHD-AFC. **F**. Enzymatic activity of caspase-3 was measured in cell lysates using a fluorogenic substrate, Ac-DEVD-AMC. The data shown represent the mean ±SD of at least three independent experiments, performed in triplicate. *P < 0.05, statistically significant relative to control (37°C).

Then, we examined the influence of heat stress on the release of cytochrome C. The Western blots showed a significant increase in the release of cytochrome C into the cytosol induced by heat stress, which increased in a time-dependent manner beginning in 3h and lasting up to 24h after heat stress (Figure 6A, 6B). Moreover, the abundance of Bax protein increased in a time-dependent fashion and with a similar increased expression trend as pro-apoptotic cytochrome C (Figure 6C). Conversely, the expression of anti-apoptotic Bcl-2 decreased in the same time frame (Figure 6C), suggesting an increase in the Bax/Bcl-2 ratio, which might be involved in apoptosis induced by heat stress (Figure 6D).

Cytochrome C interacts with apoptotic protease-activating factor-1(Apaf-1) in the cytosol resulting in the downstream recruitment, procession, and activation of pro-caspase-9 in the presence of dATP or ATP [8]. Caspase-9 cleaves and activates pro-caspase-3 and pro-caspase-7, and serves as an activating protein in the mitochondrial apoptosis pathway [7–9]. As shown in Figure 6E and 6F, caspase-9 activity significantly increased at 3h and peaked at 6h after heat stress. Caspase-3 expression was closely correlated with increased expression of caspase-9 activity.

### Cathepsin B activation of the mitochondrial apoptosis pathway in SW480 cells

**Figure 7:**
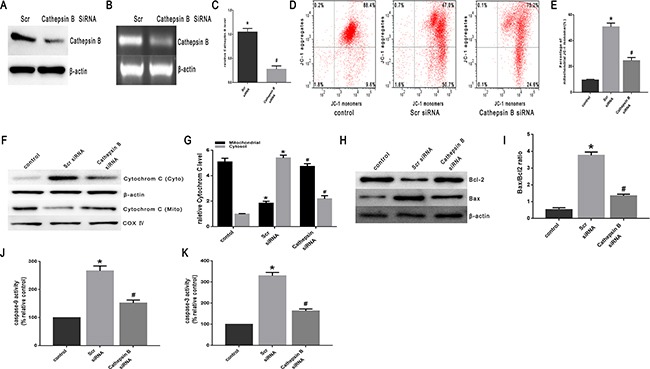
Heat stress activates apoptosis in cathepsin B siRNA-transfectant SW480 Cells SW480 cells were transfected with scrambled siRNA(Scr)or cathepsin BsiRNA (cathepsin B). **A**. Western blots of cathepsin B protein expressed in SW480 transfectant cells. β-actin was run as an internal control. **B**. RT-PCR analysis of cathepsin B in SW480 transfectant cells. Total RNA was isolated from SW480 transfectant cells. Cathepsin B mRNA levels were determined by RT-PCR and analyzed on agarose gel electrophoresis. β-actin from the same samples was amplified as control. **C**. Quantification of RT-PCR for cathepsin B after heat stress. (D-K) SW480 transfectant cells were treated with 43°C for 2h and then further incubated at 37°C for 6h; **D**. Flow cytometry analysis heat stress-induced mitochondrial depolarization (low *ΔΨ*m). **E**. The histogram represents the quantification of the percentage of low *ΔΨ*m analyzed by flow cytometry. **F**. Intracellular location of cytochrome C was determined by Western blots. β-actin was run as an internal control. COX IV was used as a mitochondrial loading control. **G**. Quantification of Western blots for cytochrome C after heat stress. **H**. Bcl-2 and Bax were determined by Western blots. β-actin was run as an internal control. **I**. Quantification of Western blots for Bax/Bcl-2 ratio after heat stress. **G**. Enzymatic activity of caspase-9 was measured in cell lysates using afluorogenic substrate, Ac-LEHD-AFC. **K**. Enzymatic activity of caspase-3 was measured in cell lysates using a fluorogenic substrate, Ac-DEVD-AMC. The data shown represent the mean ±SD of at least three independent experiments, performed in triplicate. *P < 0.05, statistically significant relative to control (37°C), ^#^*P* < 0.05, statistically significant relative to scrambled siRNA-transfected cells exposed to heat stress.

Lysosomal cysteine protease cathepsin B plays an important role in apoptosis [20]. Cathepsin B can initiate apoptosis through activating caspases, a process related to the release of Bcl-2 and cytochrome C proteins and the activation of caspase-3 [15, 20, 21]. To investigate the involvement of cathepsin B in heat stress-induced apoptosis, the effects of the transfection of cathepsin B siRNA on the cellular features of apoptosis were studied. The transfected cells were assayed for cathepsin B protein expression by Western blot assay (Figure 7A). Of the several tested siRNA target sequences, one siRNA was effective in decreasing protein levels. Cathepsin B levels measured by RT-PCR were significantly decreased in cathepsin B-transfected cells compared to control cells (scrambled siRNA) (Figure 7B, 7C). The cathepsin B siRNA-transfected cells expressed reduced levels of cathepsin B protein, and were less susceptible to heat stress-induced mitochondrial depolarization compared with control cells (Figure 7D, 7E). Compared with control cells, cathepsin B siRNA-transfected SW480 cells showed a drastic reduction in the release of cytochrome C (Figure 7F, 7G), caspase-9 activity (Figure 7J) and caspase-3 activity (Figure 7K). Meanwhile, compared to control cells, the expression of Bcl-2 was up-regulated, whereas the expression of Bax was down-regulated (Figure 7H, 7I).

### Effect of antioxidant NAC on heat stress-induced lysosomal membrane permeabilization, cathepsin B release, and apoptosis *in vitro* and *in vivo*

**Figure 8:**
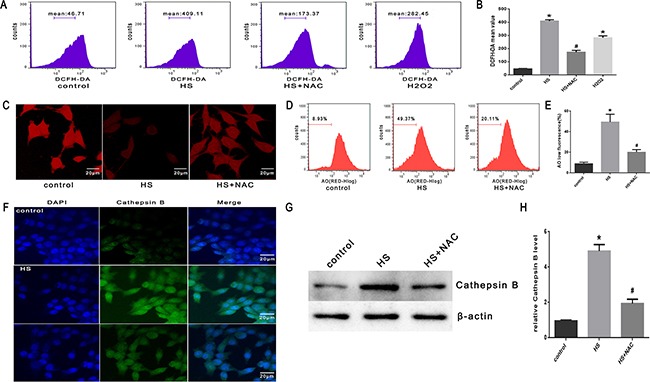
Effect of antioxidant NAC on heat stress-induced lysosomal membrane permeabilization and cathepsin B release in SW480 Cells SW480 cells were pretreated with or without 2mMN-acetyl-L-cysteine (NAC) followed by incubation at43°C for 2h and further incubated at 37°C for 6h. **A**. Flow cytometry analysis heat stress-induced ROS. **B**. The histogram represents the quantification of ROS generation analyzed by flow cytometry. **C**. Confocal laser scanning microscopy images of fluorescently labeled cells (×400). **D**. Flowcytometry analysis to count cells with a reduced number of intact lysosomes (‘pale cells’). **E**. The histogram represents the quantification of number of ‘pale cells’ analyzed by flow cytometry. **F**. Localization of cathepsin B was visualized by immunofluorescence using a confocal microscope (x200). **G**. Expression of cathepsin B was determined by Western blot. β-actin was run as an internal control. **H**. Quantification of Western blots for cathepsin B. The data shown represent the mean ±SD of at least three independent experiments, performed in triplicate. *P < 0.05, statistically significant relative to control (37°C),^#^*P* < 0.05, statistically significant relative to heat stress group (HS).

**Figure 9:**
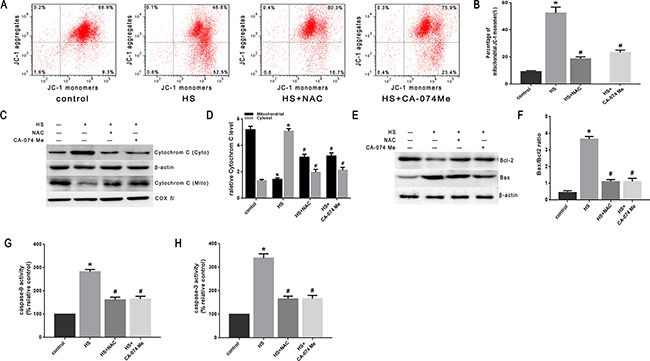
Effect of antioxidant NAC and cathepsin B inhibitor CA-074 Me on the mitochondrial apoptosis pathway in SW480 cells SW480 cells were pretreated with or without 2mM N-acetyl-L-cysteine (NAC) or 50μM CA-074 Me followed by exposure to43°C for 2h and further incubation at 37°C for 6h. **A**. Flow cytometry analysis heat stress-induced mitochondrial depolarization (low *ΔΨ*m). **B**. The histogram represents the quantification of the percentage of low *ΔΨ*m analyzed by flow cytometry. **C**. Intracellular location of cytochrome C was determined by Western blots. β-actin was run as an internal control. COX IV was used as a mitochondrial loading control. **D**. Quantification of Western blots for cytochrome C. **E**. Bcl-2 and Bax were determined by Western blots. β-actin was run as an internal control. **F**. Quantification of Western blots for Bax/Bcl-2 ratio. **G**. Enzymatic activity of caspase-9 was measured in cell lysates using a fluorogenic substrate, Ac-LEHD-AFC. **H**. Enzymatic activity of caspase-3 was measured in cell lysates using a fluorogenic substrate, Ac-DEVD-AMC. The data shown represent the mean ±SD of at least three independent experiments, performed in triplicate. *P < 0.05, statistically significant relative to control (37°C),^#^*P* < 0.05, statistically significant relative to heat stress group (HS).

To confirm the role of ROS in heat stress-induced lysosomal membrane permeabilization, cathepsin B release, and cell apoptosis, the SW480 cells were pretreated with a well-known antioxidant (NAC) that can inhibit ROS generation. As shown in Figure 8A & 8B, NAC significantly inhibited heat stress-induced ROS formation. When cells were pretreated with NAC, it promoted the recovery of lysosome stability, indicated by a much brighter fluorescence (Figure 8C) and decreased percentage of ‘pale cells’ (Figure 8D, 8E) compared to no treatment with NAC. After NAC treatment, cathepsin B fluorescence in the cytosol was reduced (Figure 8F) and the expression levels of cathepsin B induced by heat stress were down-regulated (Figure 8G, 8H). The depletion of ROS by the antioxidant NAC significantly decreased the heat stress-mediated mitochondrial depolarization (Figure 9A, 9B), cytochrome C release from mitochondria (Figure 9C, 9D), Bax/Bcl-2 ratio (Figure 9E, 9F), caspase-9 activity (Figure 9G), and caspase-3 activity (Figure 9H).

**Figure 10:**
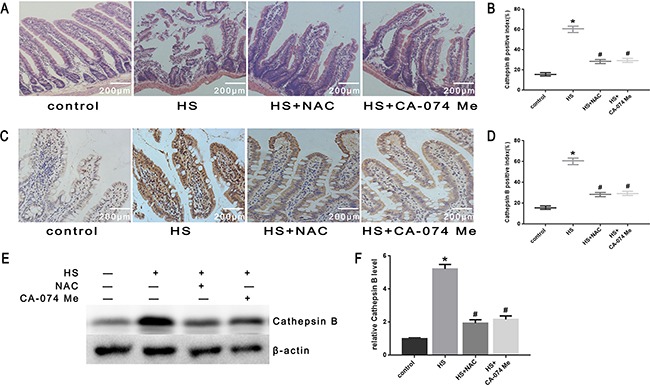
Effect of antioxidant NAC and cathepsin B inhibitor CA-074 Me in intestinal tissue injury and cathepsin B release *in vivo* BALB/c mice were pretreated with or without100mg/kg N-acetyl-L-cysteine (NAC, intraperitoneal) at 1h or 10mg/kg 50μM CA-074 Me (intraperitoneal) at 30min followed by exposure in a temperature-controlled chamber (ambient temperature 35.5±0.5°C and 60±5% relative humidity)until the rectal core temperature (Tc) reached 42°C. **A**. Pathological changes in the ileum of mice (magnification, ×200). **B**. Chiu scores of intestinal injury in mice. **C**. Cathepsin B was localized by immunohistochemical stain (magnification, ×200). **D**. Quantification of immunohistochemical stain for Cathepsin B. **E**. Expression of cathepsin B was determined by Western blot. β-actin was run as an internal control. **F**. Quantification of Western blots for cathepsin B.*P < 0.05, statistically significant relative to control (37°C),^#^*P* < 0.05, statistically significant relative to heat stress group (HS), (n=6/group).

**Figure 11:**
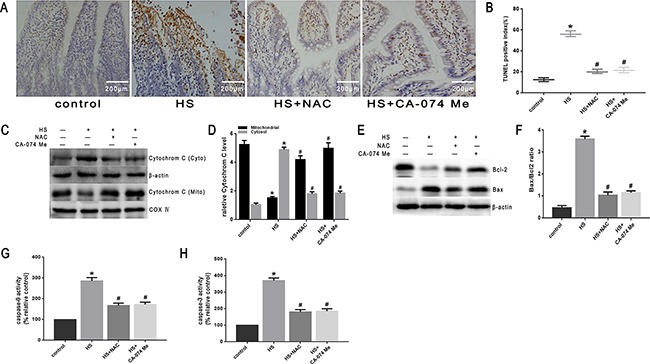
Effect of antioxidant NAC and cathepsin B inhibitor CA-074 Me in mitochondrial apoptosis pathway *in vivo* BALB/c mice were pretreated with or without100mg/kg N-acetyl-L-cysteine (NAC, intraperitoneal) at 1h or 10mg/kg 50μM CA-074 Me (intraperitoneal) at 30min followed by exposure in a temperature controlled chamber (ambient temperature 35.5±0.5°C and 60±5% relative humidity)until the rectal core temperature (Tc)reached 42°C. **A**. TUNEL staining of the ileum of mice (magnification, ×200). **B**. Quantification of TUNEL-positive index. **C**. Cytochrome C was determined by Western blots. β-actin was run as an internal control. COX IV was used as a mitochondrial loading control. **D**. Quantification of Western blots for cytochrome C. **E**. Bcl-2 and Bax were determined by Western blots. β-actin was run as an internal control. **F**. Quantification of Western blots for Bax/Bcl-2 ratio. **G**. Enzymatic activity of caspase-9 was measured in cell lysates using a fluorogenic substrate, Ac-LEHD-AFC. **H**. Enzymatic activity of caspase-3 was measured in cell lysates using a fluorogenic substrate, Ac-DEVD-AMC. *P < 0.05, statistically significant relative to control (37°C),^#^*P* < 0.05, statistically significant relative to heat stress group (HS),(n=6/group).

To further explore the mechanism of intestinal damage and the effect of ROS *in vivo*, mice were pretreated with NAC. As shown in Figure 10A, histopathologic examination was performed on the small intestine (ileum). In the control group that did not undergo heat stress, no marked damage was observed in the small intestinal mucosa. In the heat stroke group, marked villous stroma broadening, focal necrosis, and some epithelial cell detachment accompanied by marked edema and congestion were observed on histologic evaluation with H&E staining. The Chiu score was also increased (Figure 10B). After NAC treatment, we observed reduced heat stroke-induced intestinal damage and decreased Chiu scores (Figure 10A, 10B). NAC also reduced the expression of cathepsin B in the small intestine tissue analyzed by immunohistochemical stain (Figure 10C, 10D) and Western blot (Figure 10E, 10F). In the mice pretreated with NAC, there were reduced levels of apoptosis (Figure 11A, 11B), cytochrome C release from mitochondria (Figure 11C, 11D), Bax/Bcl-2 ratios (Figure 11E, 11F), caspase-9 activity (Figure 11G) and caspase-3 activity in the tissue from the small intestine (Figure 11H).

### Effect of cathepsin B inhibitor on heat stress induced apoptosis *in vitro* and *in vivo*

To further verify that cytosol cathepsin B accumulation is associated with heat stress-induced cell apoptosis, we administered a highly selective cathepsin B inhibitor (CA-074 Me). As shown in Figure 10C-10F, after pretreatment with CA-074 Me there was a significant reduction *in vivo* of cytosolic heat stress-induced cathepsin B on immunohistochemical stain (Figure 10C, 10D) and Western blot (Figure 10E, 10F). Meanwhile, pretreatment with CA-074 Me was shown to reduce intestinal tissue injury (Figure 10A), decreased Chiu score (Figure 10B), and decrease the degree of apoptosis (Figure 11A, 11B) caused by heat stroke. In addition, we found that a significant decrease in cytosolic cytochrome C (Figure 9C, 9D and Figure 11C, 11D), Bax/Bcl-2 ratio (Figure 9E, 9F and Figure 11E, 11F), caspase-9 (Figure 9G and Figure 11G) and caspase-3 activation (Figure 9H and Figure 11H) were observed both *in vitro* and *in vivo*. Mitochondrial depolarization was also alleviated in cells SW480 pretreated with CA-074 Me compared with control cells (Figure 9A, 9B).

## DISCUSSION

The small intestine plays important roles in providing a barrier to pathogens and promoting nutrient absorption. However, these functions can be compromised by heat stroke [25, 26]. Reduced blood flow to the small intestine following heat stroke occurs to preserve flow to essential organs, such as the brain and the heart [5, 12, 13]. This process greatly impairs the epithelial cells of the small intestinal villi, induces excessive apoptosis of epithelial cells, and leads to an increased risk of morbidity and mortality [12, 13]. In the current study, we found that heat stroke induced morphological injuries in mice, including villous stroma broadening, focal necrosis, and epithelial cell detachment accompanied by marked edema and congestion. We also demonstrated through TUNEL analysis that heat stroke can induce an abundance of apoptosis in tissue from the small intestine.

Mitochondria are known as the central integrators and transducers for pro-apoptotic signals, forming the nexus between the non-specific inducer phase and the final execution phase of apoptosis [8]. The central and vital event in the mitochondrial apoptotic pathway is mitochondrial outer membrane permeabilization (MOMP), which leads to the release of cytochrome C [20]. In the cytosol, Apaf-1binds cytochrome C and induces oligomerization and assembly of the apoptosome. This process then activates initiator caspase-9 by dimerization [7, 8, 20]. Activated caspase-9 proteolytically activates executioner caspases-3 and -7. This step is further facilitated by the release of apoptogenic factors from mitochondria [7, 20]. Mitochondrial integrity is also controlled by the B-cell lymphoma-(Bcl-2) family of proteins; these proteinsplay a major role in regulating mitochondrial integrity [27]. Consistently, our results demonstrated that heat stress results in a significant increase in cytochrome C release from mitochondria, Bax/Bcl-2 ratio, caspase-9 activity, and caspase-3 activity in SW480 cells and small intestinal tissue. In addition, our results also revealed that mitochondrial depolarization (low *ΔΨ*m) was increased after heat stress in SW480 cells. Therefore, our results suggested that heat stress might mediate intestinal tissue and epithelial cell damage through the activation of the mitochondrial apoptotic pathway.

Lysosomes and lysosomal proteases are key components of various cell death pathways, including apoptosis, autophagy and necrosis [15, 20, 21]. More recently, research has explored the possible interplay between the lysosomal apparatus and mitochondria to create a “lysosomal–mitochondrial axis” apoptotic signaling pathway [17–19]. Lysosomal membrane permeabilization is considered the critical step in this pathway, and has been shown to coincide with the relocation of lysosomal enzymes, such as cathepsins B and D to the cytosol [15, 20, 21]. Activation of cathepsin B can induce apoptosis in cells, possibly leading to a downstream activation of caspases and subsequent mitochondrial depolarization [15, 20, 21]. Cathepsin B plays such a vital role in the process that the lysosomal–mitochondrial axis theory of cell death has been proposed as a kind of cathepsin B-regulated apoptosis [15, 17]. In this report, lysosomal membrane permeabilization, the critical step in the lysosomal–mitochondrial apoptosis pathway, was found to occur when the SW480 cells were exposed to heat stress. Heat stress-induced cathepsin B release into the cytosol occurred both in SW480 cells and in intestinal tissue. We also demonstrated how cathepsin B siRNA-transfected cells were less susceptible to heat stress-induced mitochondrial depolarization (low *ΔΨ*m). Compared to control cells, cathepsin B siRNA-transfected SW480 cells displayed a drastic reduction in the release of cytochrome C, caspase-9 and caspase-3; meanwhile, the expression of Bcl-2 was up-regulated, whereas the expression of Bax was down-regulated. The importance of these lysosomal proteases during apoptosis was examined further using lysosomal cysteine and serine protease inhibitor CA-074 Me both *in vitro* and *in vivo*. The cathepsin B inhibitor could partially inhibit the release of cathepsin B and cytochrome C, moderate the increase in the Bax/Bcl-2 ratio, and preventcaspase-9 and caspase-3 activation induced by heat stress both *in vitro* and *in vivo*. Taken together, it is plausible that heat stress-induced lysosomal membrane permeabilization and cathepsin B release into the cytosol mediated the downstream lysosomal–mitochondrial apoptotic pathway both in intestinal tissue and cultured epithelial cells.

Oxidative stress has been associated with many forms of programmed cell death. There exists strong evidence that ROS are involved in the process of apoptosis [17]. Our previous studies have found that heat stress-mediated oxidative stress works primarily by increasing the production of ROS, which act as upstream signals to promote the early steps of apoptosis from heat stress in HUVEC cells [7, 8]. Oxidative stress during apoptosis and lysosomal involvement in the apoptotic process is being increasingly recognized as a cell signaling mechanism; it has also been previously demonstrated that ROS can cause lysosomal membrane permeabilization [15, 17]. Our study demonstrates that heat stress-induced ROS increased immediately in SW480 cells after heat stress. Furthermore, the use of NAC, an agent that inhibits ROS generation, was able to block the movement of cathepsin B to the cytosol, inhibit the release cytochrome C, moderate the Bax/Bcl-2 ratio, and prevent caspase-9 and caspase-3 activation mediated by heat stress both *in vitro* and *in vivo*. Taking the data in aggregate, the heat stress-induced production of ROS appear to act as an upstream signal that triggers lysosomal membrane permeabilization and cathepsin B release to cytosol, thus regulating he lysosomal–mitochondrial pathway and promoting apoptosis from heat stress both in intestinal tissue and in epithelial cells.

In conclusion, our data suggest that both intestinal tissue and epithelial cells undergo apoptosis soon after heat stress. Heat stress-induced apoptosis is associated with the lysosomal–mitochondrial apoptosis pathway by causing lysosomal membrane permeabilization and cathepsin B release into the cytosol. This process appears to be mediated by ROS generation both *in vitro* and *in vivo*. These findings may offer potentially targets to combat heat stress and provide strategies to repair intestinal epithelial tissue injured by heat stroke.

## MATERIALS AND METHODS

### Cell culture and treatments

SW480 cells were grown in culture medium as instructed by the manufacturer. Cell culture dishes containing SW480 cells were sealed with Parafilm and immersed for 2h in a circulating water bath thermo-regulated at 37±0.5°C(control) or at 43°C. Culture medium was then replaced with fresh medium. The cells were further incubated at 37°C for additional time (0h, 1h, 3h, 6h, 12h or 24h). SW480 cells were pretreated with or without 2mM N-acetyl-L-cysteine (NAC) [17] or 50μM CA-074 Me [28] followed by further incubation at 43°C for 2h and then at 37°C for 6h.

### Cell viability assays

Cell proliferation was assessed using the Premixed WST-1 Cell Proliferation Reagent (Clontech Laboratories Inc., Mountain View, CA, USA) as stated in the manufacturer's instructions. Lactate dehydrogenase (LDH) enzymatic activity was assayed using a commercially available kit (JianChen Co, Nanjing, China).

### Measurements of ROS

To analyze the kinetics of ROS generation, cells were heat stressed at 43°C for 2hand further incubated at 37°C for the above indicated times. ROS were detected using the fluorescent probe DCFH-DA (Molecular Probes). SW480 cells were incubated in the dark with 10μM DCFH-DA for 30 min at 37°C. DCFH-DA oxidized by ROS produces DCF with green fluorescence. Hydrogen peroxide (H_2_O_2_) was used as a positive control. The fluorescence intensity generated by the ROS probes was analyzed by flow cytometric analysis.

### Lysosomal stability assessment

Lysosomal membrane permeabilization (LMP) was induced by heat stress and analyzed using the acridine orange (AO) relocation method. Cells were heat stressed at 43°C for 2h and further incubated at 37°C for the indicated times. Cells were labeled with 5μM AO for 30 min at 37°C in the dark. The fluorescence intensity of the AO probes was analyzed by flow cytometric analysis. Images were captured using laser scanning confocal microscopy.

### Measurement of mitochondrial membrane potential

The mitochondrial membrane potential (*ΔΨm*) was measured using the fluorescent probe JC-1 (Invitrogen, CA, USA). In mitochondria with normal membrane potentials, JC-1 forms aggregates that fluoresce red, whereas in damaged, depolarized mitochondria, JC-1 forms monomers that fluoresce green. Cells were heat stressed at 43°C for 2h and further incubated at 37°C for the indicated times. Cells were then incubated in DMEM containing 5μmol/L JC-1 at 37°C for 15 min. Relative fluorescence was subsequently measured by flow cytometry (BD FACS Verse™; BD, USA). Data were analyzed using BD FAC Suite software.

### siRNA transfection

Small-interfering RNA (siRNA) for cathepsin B was designed and synthesized by Guangzhou RiboBio (RiboBioInc, China). The sequence of each gene and their scrambled form are shown asfollows:1) cathepsin B, sense and antisense siRNAs were 5’- GGCCCCCTGCATCTATCG -3’ and 5’- AGGTCTCCCGCTGTTCCACTG -3’, respectively; 2) the scrambled control, sense and antisense siRNAs were 5’ -UGGUUUACAUGUCGACUAA(dTdT)-3’and 5’-UUAGUCGACAUGUAAACCA(dTdT)-3’, respectively. Twenty-four hours prior to transfection, SW480cells were plated on a 6-well plate (Nest, Biotech, China) at 30–50% confluence. Cells were then transfected using TurboFectTM siRNA Transfection Reagent (Fermentas, Vilnius, Lithuania) following the manufacturer's protocol. Cells were collected after 48-72 h. The mRNA and protein levels of the SW480cells were estimated by RT-PCR and Western blotting. After incubating for 2days at 37°C in a humidified atmosphere of 5% CO_2_, the cells were exposed to heat stress.

### Cathepsin B immunofluorescence analysis

Cells were grown on 35-mmglass coverslips. After treatment, the cells were washed once in PBS, fixed in ice-cold methanol at 4°Cfor 6 min, and permeabilized with0.3% Tween 20 in PBS for 3 min at room temperature. After being washed with PBS, the cells were incubated in blocking buffer (20% goat serum, 0.05% Tween 20 in PBS) for 1h at 37°C, then incubated overnight at room temperature with mouse anti-human cathepsin B antibody (dilution 1:500 in 5% goat serum, 0.05% Tween 20 in PBS). The cells were rinsed with 0.05% Tween 20 in PBS, incubated with Alexa Fluor 488-conjugated anti-mouse IgG (dilution 1:500 in 1% BSA,0.05% Tween 20 in PBS) for 1 h at 37°C, mounted by using Prolong Anti fade kit (Molecular Probes), and visualized by using an inverted laser-scanning confocal microscope (Model 510; Carl Zeiss, Jena, Germany) with excitation and emission wavelengths of 488 nm and 507 nm, respectively.

### Animals

Pathogen-free 6- to 8-week old male BALB/c mice were housed individually under controlled environmental conditions with a 12-hour light/dark cycle and unrestricted access to pellet food and water throughout the study. The animals were purchased from the Experimental Animal Center of the Southern Medical University in Guangzhou, P.R. China (Certification: SCXK (Guangzhou) 2011-0015). All efforts were made to reduce the number of animals used and to minimize animal discomfort. The experimental protocols were approved by the Animal Care and Use Committee of the Southern Medical University, Guangzhou, China. None of the authors are members of this committee. The care of the animals was in accordance with the National Institutes of Health Guidelines as well as with those of the Chinese National one.

### Experimental groups and drug administration

Mice were fasted for 12h prior to the experiment, but were allowed water adlibitum. After being stabilized for 6h at an ambient temperature (25±0.5°C) with a humidity of 35±5%, mice were pretreated with or without 100mg/kg N-acetyl-L-cysteine (NAC, intraperitoneal) at 1h or 10mg/kg CA-074 Me (intraperitoneal) at 30min [30] before heat stress. Then the mice were divided into four groups: the control group and the heat stress (HS) group, HS+ NAC group and HS+ CA-074 Me group. The animals in the HS group, HS+ NAC group and HS+ CA-074 Me group were placed in a pre-warmed incubator maintained at35.5±0.5°C with a relative humidity of 60±5% in the absence of food and water. The animals in the control group were sham-heated at a temperature of 25±0.5°C and a humidity of 35±5% for a time comparable to that of the HS group. The rectal core temperature (Tc) was continuously monitored with a rectal thermometer.

### Histopathological analysis

Mice were anesthetized by intraperitoneal injection of urethane and sacrificed. Samples of ileum were quickly excised, sliced into transverse or longitudinal sections, and fixed in 10% neutral-buffered formalin. The tissues were then embedded in paraffin blocks, and serial sections were stained with hematoxylin and eosin for microscopic evaluation at a magnification of ×200. Morphological changes were assessed and graded in a blinded manner by two certified veterinary pathologists using the intestinal injury score developed by Chiu et al.

### Immunohistochemical staining

Immunohistochemical staining was performed as previously described. Samples of ileum were quickly excised and immediately immersed in 4% paraformal dehyde for over 24h at 4°C. Sections of ileum (5μm thick) were blocked in 3% H_2_O_2_ and 3% normal goat serum and incubated overnight with anti-mouse cathepsin B polyclonal antibodies (1:200, Abcam, England). The secondary antibodies, secondary biotinylated conjugates and diaminobenzidine, were obtained from the GTVision™ III SP rabbit/mouse Horseradish Peroxidase (HRP) kit (DAB) (Dako, Denmark). An examiner blinded to the experimental groups randomly counted the cells labeled with cathepsin B throughout five lesion regions in the injured-side cortex under a light microscope at ×200.

### TUNEL analysis

Terminal deoxynucleotidyl transferase-dUTP nick-end labeling (TUNEL) staining was used to detect apoptotic cells with a TUNEL staining kit (Roche, Basel, Switzerland) according to the manufacturer's instructions. For both immunohistochemistry and TUNEL staining, two slides (at least 200 lmapart) per rat with six rats per group were used. On each slide, two microvessels in the frontal cortex were randomly selected. TUNEL-positive cells were identified, counted, and analyzed with the help of an investigator who was blinded to the experimental treatments. The data are presented as the percentages of TUNEL positive.

### Western blot analysis

Western blot analysis was performed as described previously using the following antibodies [17, 28]: cathepsin B, cytochrome C, Bcl-2 and Bax (all used at 1:1000; Abcam). An HRP-conjugated anti-rabbit IgG antibody was used as the secondary antibody (Zhongshan Inc, China). Signal was visualized with enhanced chemiluminescence (Pierce, Rockford, IL, USA).

### Caspase activity assay

Cells or samples of ileum were harvested and lysed. Cell lysates were first incubated at -80°C for 30 min and subsequently incubated at 37°Cwith the appropriate caspase substrates using a Quadruple Monochromator Microplate Reader (Infinite M1000, Tecan US, NC, USA). Caspase activities were measured by cleavage of the following fluorogenic peptide substrates: Ac-LEHD-AFC (caspase-9) and Ac-DEVD-AMC(caspase-3). Caspase activity is represented as relative cumulative fluorescence of the kinetic reaction relative to untreated controls.

### Statistical analysis

All data were analyzed for statistical significance using SPSS 13.0 software (SPSS, Chicago, IL, USA). Data were expressed as mean ± SD from at least 3 independent experiments performed in duplicate. Statistical comparisons of the results were performed using one-way analysis of variance (ANOVA). A p-value<0.05 was considered to be statistically significant.
